# Central Thalamic Deep-Brain Stimulation Alters Striatal-Thalamic Connectivity in Cognitive Neural Behavior

**DOI:** 10.3389/fncir.2015.00087

**Published:** 2016-01-13

**Authors:** Hui-Ching Lin, Han-Chi Pan, Sheng-Huang Lin, Yu-Chun Lo, Elise Ting-Hsin Shen, Lun-De Liao, Pei-Han Liao, Yi-Wei Chien, Kuei-Da Liao, Fu-Shan Jaw, Kai-Wen Chu, Hsin-Yi Lai, You-Yin Chen

**Affiliations:** ^1^Department and Institute of Physiology, School of Medicine, National Yang Ming UniversityTaipei, Taiwan; ^2^Brain Research Center, National Yang Ming UniversityTaipei, Taiwan; ^3^Institute of Neuroscience, National Yang Ming UniversityTaipei, Taiwan; ^4^Department of Neurology, Tzu Chi General Hospital, Tzu Chi UniversityHualien, Taiwan; ^5^Institute of Biomedical Engineering, National Taiwan UniversityTaipei, Taiwan; ^6^Institute of Medical Device and Imaging, National Taiwan University College of MedicineTaipei, Taiwan; ^7^Centre for Life Sciences, Singapore Institute for Neurotechnology, National University of SingaporeSingapore, Singapore; ^8^Institute of Biomedical Engineering and Nanomedicine, National Health Research InstitutesMiaoli, Taiwan; ^9^Department of Biomedical Engineering, National Yang Ming UniversityTaipei, Taiwan; ^10^Graduate Institute of Biomedical Electronics and Bioinformatics, National Taiwan UniversityTaipei, Taiwan; ^11^Interdisciplinary Institute of Neuroscience and Technology, Qiushi Academy for Advanced Studies, Zhejiang UniversityHangzhou, China

**Keywords:** deep brain stimulation, reward-associated learning, thalamus, local field potentials, functional connectivity

## Abstract

Central thalamic deep brain stimulation (CT-DBS) has been proposed as an experimental therapeutic approach to produce consistent sustained regulation of forebrain arousal for several neurological diseases. We investigated local field potentials (LFPs) induced by CT-DBS from the thalamic central lateral nuclei (CL) and the striatum as potential biomarkers for the enhancement of lever-pressing skill learning. LFPs were simultaneously recorded from multiple sites in the CL, ventral striatum (Vstr), and dorsal striatum (Dstr). LFP oscillation power and functional connectivity were assessed and compared between the CT-DBS and sham control groups. The theta and alpha LFP oscillations were significantly increased in the CL and striatum in the CT-DBS group. Furthermore, interhemispheric coherences between bilateral CL and striatum were increased in the theta band. Additionally, enhancement of *c-Fos* activity, dopamine D2 receptor (Drd2), and α4-nicotinic acetylcholine receptor (α4-nAChR) occurred after CT-DBS treatment in the striatum and hippocampus. CT-DBS strengthened thalamic-striatal functional connectivity, which demonstrates that the inter-regional connectivity enhancement might contribute to synaptic plasticity in the striatum. Altered dopaminergic and cholinergic receptors resulted in modulation of striatal synaptic plasticity's ability to regulate downstream signaling cascades for higher brain functions of lever-pressing skill learning.

## Introduction

Deep brain stimulation (DBS) is a potent therapeutic approach of electrical stimulation through electrodes implanted in specific regions to modulate abnormal neuronal activities that contribute to neurological diseases and psychiatric disorders (Kolb et al., 1983; Overbeek et al., 2013; Williams and Okun, 2013; Schlaepfer and Bewernick, 2014). Several studies demonstrated DBS can modulate the firing patterns of neurons through changes in subregional synchronization and low-frequency rhythmic oscillation (Bergman et al., 1998; Vitek and Giroux, 2000; Deuschl et al., 2001). Studies have shown that DBS mediates neurological changes and behavioral improvement, and interval stimulation of the medial temporal lobe and the memory formation-related region with specific frequencies and critical timing is important for memory processing (Suthana et al., 2012; Fell et al., 2013; Lee et al., 2013).

Recently, several animal experiments and clinical trials have indicated that DBS contributed to enhanced learning and memory (Suthana and Fried, 2014). Applications are being developed for memory impairment due to Alzheimer disease, traumatic brain injury, temporal lobe epilepsy, stroke, and encephalitis. Many studies (Halgren et al., 1985; Lacruz et al., 2010; Stone et al., 2011) found that electrical stimulation to the hippocampal entorhinal cortex circuit (Squire et al., 2004), that has been shown to improve spatial learning. However, hippocampal DBS has been found to disrupt memory (Ego-Stengel and Wilson, 2010) and decline learning (Leung and Shen, 2006) due to electrical stimulation induced seizures.

Central thalamus (CT) nuclei contained densely populated with neurons that widely project to striatum as well as cortical targets and collectively provide the largest thalamic efference to the striatum, which are hypothesized to synchronize activity in neural networks that underlie cognitive functions (Mengual et al., 1999; Jones, 2009). Meanwhile, CT nuclei regulated arousal and awareness, influencing activity in distributed neural networks that give widespread effects on cortical and subcortical functions (Parikh and Sarter, 2008; Robbins and Arnsten, 2009). Alternatively, CT of investigation is whether DBS may be a potent therapeutics for disorders of learning and memory. Several studies have demonstrated that thalamic DBS is a safe and efficacious treatment for essential tremor (Flora et al., 2010; Baizabal Carvallo et al., 2011). It has also been reported that DBS at CT (CT-DBS) enhanced exploratory motor behaviors and cognitive performance through neocortical and hippocampal neuronal activation by specific regulation of *c-Fos* and immediate-early gene–encoded protein Egr-1 (zif268) expressions in normal rats (Shirvalkar et al., 2006). Moreover, Schiff (Schiff et al., 2007) showed that bilateral CT-DBS could restore consciousness in patients in a coma by changing the arousal state. Thus, it has been proposed that CT-DBS could be an available treatment for remediation of learning and memory deficits.

An important anatomical specialization of the CT that supports an overall role in shifting levels of activity across broad cerebral networks is their strong efference to the striatum. Deschenes et al.'s research demonstrated the neuronal projections of CT to the striatum and cortical layers, defined by biocytin anterograde labeling (Deschenes et al., 1996). The striatum is associated with numerous cognitive processes, that plays an important role in motor control (Yin and Knowlton, 2006) and reward cue-reward association tasks (Atallah et al., 2007; Jacquet et al., 2013). Striatum has been implicated in the modulation of motor control and learning ability by receiving neural signals from thalamus and transmitting to the motor cortex (Yin and Knowlton, 2006). In addition, Atallah et al. (2007) demostrated that, the ventral striatum (Vstr) is critical for skill learning, and the dorsal striatum (Dstr) is important for skill performance but not for learning.

Based on the anatomical connections of CT with the striatum, we were interested in the direct electrical stimulation of CT that altered the changes in functional connectivity for the targeted Vstr and Dstr, the stimulation site of CT, which improved the skill learning process. Therefore, spontaneous fluctuations in the local field potentials (LFPs) were used to investigate the full functional connectivity pattern of the paired brain areas. Synchronization of regional neuronal activity due to post-synaptic activation gave rise to LFP oscillations, and it played a major role in functional communication related to memory, integrative functions (Basar et al., 2001), information transfer, perception, and motor control (Fries, 2005).

In this study, we designed a water reward-related skill-learning task to explore the CT-DBS influence on cognitive performance. We performed simultaneous multi-site LFP recordings and investigate the functional connectivity in awake rats to assess the effects of CT-DBS and sham. LFP activities were recorded from bilateral CT, Vstr, and Dstr, and the stimulation sites were in bilateral CT. In addition, we identified possible molecular mechanisms by examining the protein level of dopamine and acetylcholine receptors. We hypothesized that functional connectivity could be enhanced by CT-DBS treatment in the reward and skill learning-related brain areas. Regulation of the synaptic dopaminergic and cholinergic systems are required for lever-pressing skill learning.

## Materials and methods

### Animal preparation

Twenty male adult Sprague-Dawley rats (250–300 g) were maintained on a 12-h light-dark cycle (light from 7.00 h to 19.00 h) at a constant temperature of 22 ± 3°C in the experimental animal center of National Yang Ming University. All experiments were performed in accordance with the approved guidelines and regulations, and were approved by the Institutional Animal Care and Use Committee of the National Yang Ming University. All animals were equally divided into the DBS group (*N* = 10) and sham control group (*N* = 10) to investigate the effect of CT-DBS on the animal behavioral tasks.

### Animal surgical procedures for neural implantation

The animals were anesthetized with intramuscular tiletamin and zolazepam (Zoletil 50, Virbac, Carros, France), 6 mg/kg each, suspended in 8 μg/kg Dexdomitor (Orion Pharma, Esbo, Finland). The anesthetized rats were placed in a stereotaxic frame (Model 962, Kopf Instruments, Tujunga, CA), and a craniotomy was performed over the location of electrode implantation.

In this study, an 8-channel stainless microwire electrode array (product # M177390, 30-μm diameter, California Fine Wire Co., Grover Beach, CA, USA), combined with two 1 × 4 arrays (not pictured), was used to perform CT-DBS and multi-site recording. One 1 × 4 array was geometrically designed from two pairs of microwires that were implanted bilaterally into the CL (AP: –3.5 mm; ML: ±1.4 mm; VD: 5 mm) to perform both bipolar CT-DBS and LFP recordings. The spacing between each pair of the microwires is 200 μm (Figure S1A, Supplementary Note 1). The other 1 × 4 array also was designed pair two o microwires, that was implanted into the bilateral Vstr (AP: 0.8 mm; ± ML: 2.2 mm; VD: 6.2 mm) and Dstr (AP: 0.8 mm; ± ML: 2.5 mm; VD: 3.5 mm) for LFP recordings, respectively. The spacing between each pair of microwires is 400 μm (Figure S1B, Supplementary Note 1). The spacing between the two 1 × 4 arrays was 4.3 mm in the anterior–posterior direction. A stainless steel screw was secured to the skull over the cerebellum as a reference electrode. The microwire electrode array was secured in the skull using dental acrylic and was covered with a small amount of 2% agar. One week of recovery after the implantation, we performed the behavioral tasks combined with CT-DBS and LFP recording. The implantation sites of the electrodes were confirmed and examined by Nissl staining (Figure S1C, Supplementary Note 1).

### Behavioral training

**Figure 1:**
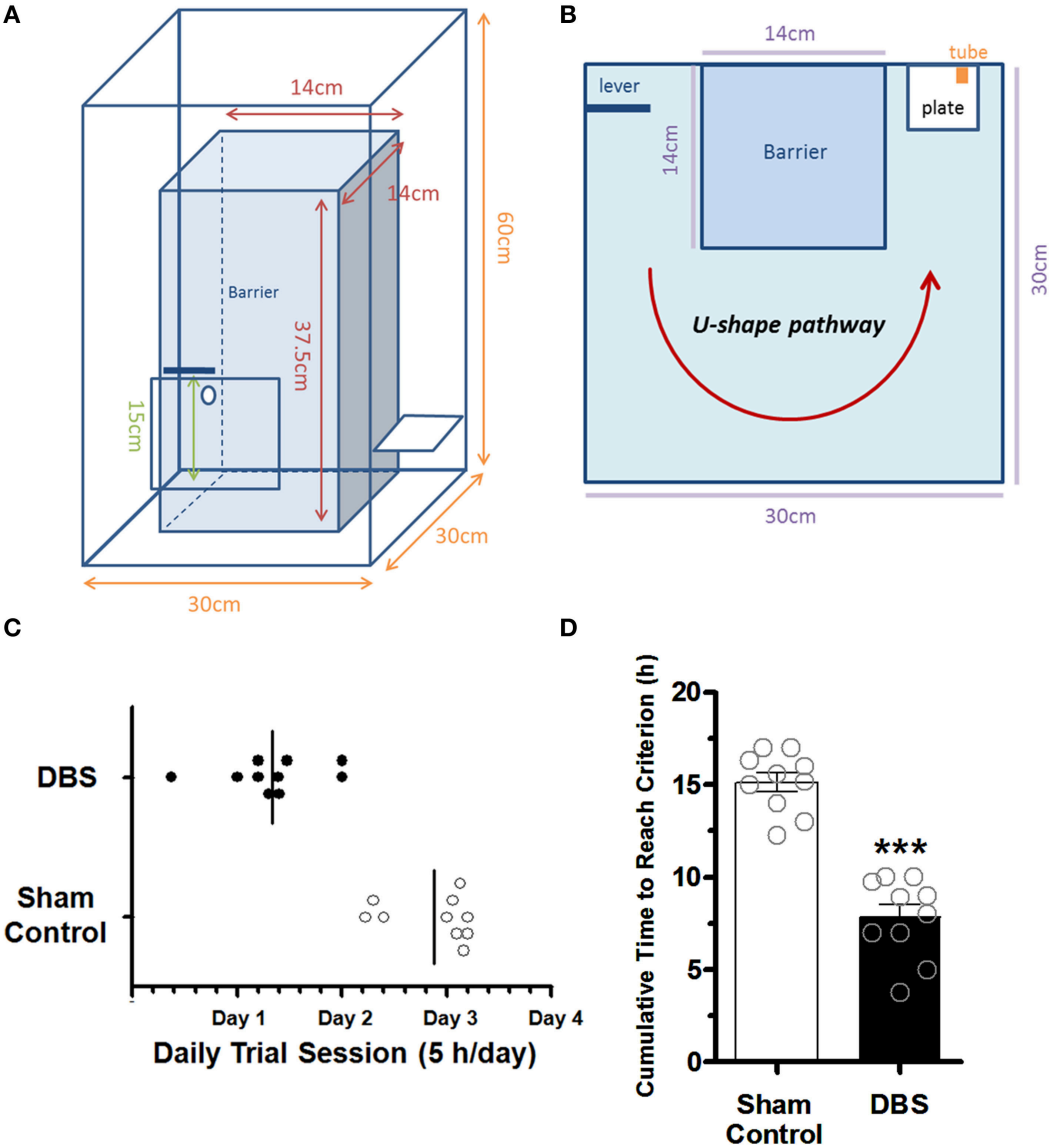
**The Plexiglas testing box and comparison of the time to reach the criterions between the sham control and CT-DBS-treated group**. The side view **(A)** and top view **(B)** of the schematics of the Plexiglas testing box. **(C)** The lotted time necessary for the rats to reach the criteria set for the water reward-related lever-pressing learning within daily trial session. **(D)** The cumulative time to reach the criterion for the water reward-related lever-pressing learning. The symbol ^***^indicates significant difference as compared with the sham control (*P* < 0.001, Wilcoxon two-sample tests, *N* = 10).

The implanted rats were single housed and deprived of water for 8 h before lever-pressing training. The lab-designed Plexiglas testing box (Figures 1A,B) used in the present study was based on *Skinner box module* (Skinner, 1992) which is known to be related to instrumental conditioning (operant conditioning; Balleine et al., 2003; Atallah et al., 2007). All implanted animals underwent LFP recording for 30 min as a baseline before the 1st reward training. Before each daily reward training, rats in the CT-DBS group received 100-Hz biphasic stimulus (0.4 mA, 25 μs per phase pulse) or the sham rats without DBS were placed in another plastic cage (30 cm diameter, 38 cm height) for 30 min. Following 30-min CT-DBS (or sham), each animal was individually introduced into the Plexiglas testing box for training the associated lever-pressing (appetitive behavior) and water-reward (instrumental skill). The cumulative time to reach the successful instrumental skill for each rat was analyzed offline by a video camera, which was placed above the Plexiglas testing box during the training sessions. In this study, the water-deprived rats had to press the lever to obtain the water conducted in a lab-designed Plexiglas testing box, that they learn the associated lever-pressing (appetitive behavior) and water-reward (consummatory behavior). We have defined the criterion for the successful skill learning was to consecutively repeat the lever-pressing and water-drinking for five times during daily 5-h sessions (9:00–14:00), for 4 days at the most. Once reaching the criterion or end of the daily training time period, LFPs were recorded for 30 min to evaluate the changes in LFP spectrum and coherence between groups. Each group was equally divided into two subgroups, and the animals were sacrificed 2 h after DBS (or sham) for further immunohistochemistry and Western blot studies.

### Neural recording and data analysis

Many studies provided evidences that the rat Vstr and Dstr play distinct roles in instrumental conditioning (skill learning; Atallah et al., 2007; Yin et al., 2008) and CT has distinct afferent and efferent connections that appear organized to project to anatomically related targets in the cerebral cortex and basal ganglia (striatum; Chen et al., 2014). In this study, multi-site LFPs were recorded bilaterally in the CL, Vstr, and Dstr using the Cerebus data acquisition system (Blackrock Microsystems, Salt Lake City, UT, USA) to explore changes of neural oscillation and functional connectivity. Neural signals were amplified, filtered at cut-off frequencies of 0.3 and 250 Hz, and sampled at 1 kHz. All data analysis was post-processed with MATLAB (R12, Mathworks Inc., Natick, MA, USA). The comparison of spectral power of LFP oscillations and coherence between DBS and sham control group were further analyzed.

LFP data for delta (1–4 Hz), theta (4–7 Hz), alpha (7–13 Hz), and beta (13–20 Hz) bands were calculated from the power spectral density (PSD), which was computed via Welch's method (see Supplementary Note 2). The coherence, the principal measure of functional connectivity used in this study (see Supplementary Note 3), provides a frequency-domain measurement of the linear magnitude and phase relationships between each channel pair of LFPs (Srinath and Ray, 2014). Intrahemispheric coherence in each hemisphere was examined for adjacent microwire electrode pairings. Interhemispheric coherence was examined for electrode pairings across the hemispheres. For comparison of functional connectivity between groups, the intrahemispheric coherence and interhemispheric coherence of each spectrum band was normalized to the percent coherence change (△Coh^intra^(site A−site B)% and △Coh^inter^(site A−site B)%), (1) subtracting the baseline coherence, and then (2) dividing the baseline coherence. The baseline was chosen as the LFP of coherence at each frequency band from before behavioral training.

### Immunohistochemistry

Ten anesthetized rats (DBS group: *N* = 5; sham control group: *N* = 5) were perfused with phosphate-buffered saline (PBS) with 0.05% heparin and 4% paraformaldehyde (PFA, Sigma-Aldrich, St. Louis, MO, USA). The rat brain was extracted from the skull and soaked in a mixture of 4% PFA and 30% sucrose (J.T. Baker, Center Valley, PA) at 4°C for 72 h, sliced on a freezing microtome at 30 μm, and stored in PBS at 4°C. The brain sections (30 μm) were washed with PBS, permeated with 0.2% triton X-100, and incubated with 3% H_2_O_2_ and 10% MeOH. Then, the brain sections were blocked with 3% normal goat serum (NGS) and hybridized with anti-*c-Fos* antibody (rabbit, 1:10000; Novus Biologicals, Littleton, CA, USA). The sections were then washed, hybridized with biotinylated goat anti-rabbit IgG antibody (1:500; Vector Laboratories, Burlingame, CA, USA), and incubated with horseradish peroxidase (HRP)-conjugated avidin complex (Vector Laboratories, Burlingame, CA, USA). In addition, the sections were incubated with 2% diaminobenzidine (DAB; Sigma-Aldrich, St. Louis, MO, USA) and 4% ammonium nickel sulfate (Sigma-Aldrich, St. Louis, MO, USA) in 0.1 M Na-K PB and developed with 0.004% H_2_O_2_.

The quantification of *c-Fos* positive immunoreactivity was performed bilaterally for 30 slices ranging from 1.2 to –3.5 mm to the bregma for each rat for each selected brain region using freeware Image Processing and Analysis in Java (ImageJ, National Institute of Health, Bethesda, MD, USA). Cell counts/mm^2^ were analyzed for the bilateral CL, primary motor cortex (M1), anterior cingulate cortex (ACC), caudate-putamen in dorsal striatum (CPu), accumbens nucleus in ventral striatum (NAc), retrosplenial cortex (Rsc), parietal association cortex (PtA), and hippocampus (CA1, CA3, and DG; Paxinos and Watson, 2005). The density of *c-Fos* positive cells for each brain area in the CT-DBS group was normalized using the mean values of the control group.

### Western blots

The striatum or hippocampus was dissected from the brain tissues of the other ten rats (DBS group: *N* = 5; sham control group: *N* = 5). Protein samples were extracted in ice-cold lysis buffer (50 mM Tris-HCl, pH = 7.5, 0.3 M sucrose, 5 mM EDTA, 2 mM sodium pyrophosphate, 1 mM sodium orthovanadate, 1 mM PMSF, 20 μg/ml leupeptin, and 4 μg/ml aprotinin) and then separated (30 μg) by SDS-PAGE, and trans-blotted onto polyvinylidene difluoride (PVDF) membranes (Millipore, Billerica, MA, USA). The membranes were hybridized with anti-dopamine D2 receptor (Drd2; 1:1000; ADR-002-50UL, Alomone Labs, Jerusalem, Israel) or anti-nicotinic acetylcholine α_4_ receptor (α4-nAChR; 1:1000; ANC-004-50UL, Alomone Labs, Jerusalem, Israel) antibodies. Then, the blots were washed and incubated with HRP-conjugated goat anti-rabbit IgG antibody (1:1000 dilution; Jackson ImmunoResearch Inc., West Grove, PA, USA), and developed by Luminata Forte Western HRP substrate (Millipore, Billerica, MA, USA). The images were recorded using the luminescence imaging system (LAS-4000, Fujifilm, Tokyo, Japan). A gel analysis plug-in for the ImageJ software was used to quantify the intensity of the protein bands.

### Statistical analysis of grouped data

Non-parametric statistical analyses between groups were tested using a Wilcoxon two-sample test. To assess performance with the water reward-related lever-pressing learning, we analyzed the effect of DBS on the changes in LFP PSD in multiple areas by averaging the power over each frequency point in multiple spectral bands, and then performing a Wilcoxon signed-rank test (*N* = 10) on PSD in each frequency band as compared with those before behavioral training.

After the water reward-related lever-pressing learning, the Wilcoxon two-sample test was used to compare the differences in LFP PSD and the changes in synchronization (coherence) between the DBS and sham control groups. The significance level was corrected to *P* < 0.0125 using a Bonferroni correction for the comparison of four bands. The comparison of *c-Fos* expression and Drd2 and α4-nAChR protein expression (Western blotting) between the groups was explored using the Wilcoxon two-sample test. A probability value of *P* < 0.05 was used as the criterion to determine statistical significance. The resulting mean values and standard error (mean ± SEM) for the data, including cumulative time to reach the successful instrumental skill, LFP spectrum and coherence, and expression of *c-Fos*, Drd2, and α4-nAChR proteins, are presented in the text.

## Results

### Behavioral task comparison: CT-DBS vs. sham control

To examine whether CT-DBS has an effect on cognitive function, we developed a water reward-related lever-pressing learning for the rats. The trained rats had to press the lever on the left side (1st action) of the box and then went along the U-shaped path to a water port on the right side of the box within 3 s to receive a reward (2nd action; Supplementary Video 1). Behavioral data showed the animals in CT-DBS group completed the lever-pressing task in 2th or 3th day while the animals in sham control group completed the lever-pressing task in 3th or 4th day as shown in Figure 1C. The learning criterion was defined by consecutively repeating the lever-press—water association more than five times during the study. Behavioral data showed animals in the DBS-treated group (7.84 ± 0.7 h) had a significantly shorter cumulative time to reach the criterion (^*^P < 0.001, Wilcoxon two-sample tests, *N* = 10) as compared with sham control animals (15.13 ± 0.5 h, *N* = 10), as shown in Figure 1D. The data suggested that the rats treated with CT-DBS had an enhanced rate of acquisition of the task of performing lever-pressing learning in comparison to sham control rats.

### Neural oscillation comparison: CT-DBS vs. sham control

**Figure 2:**
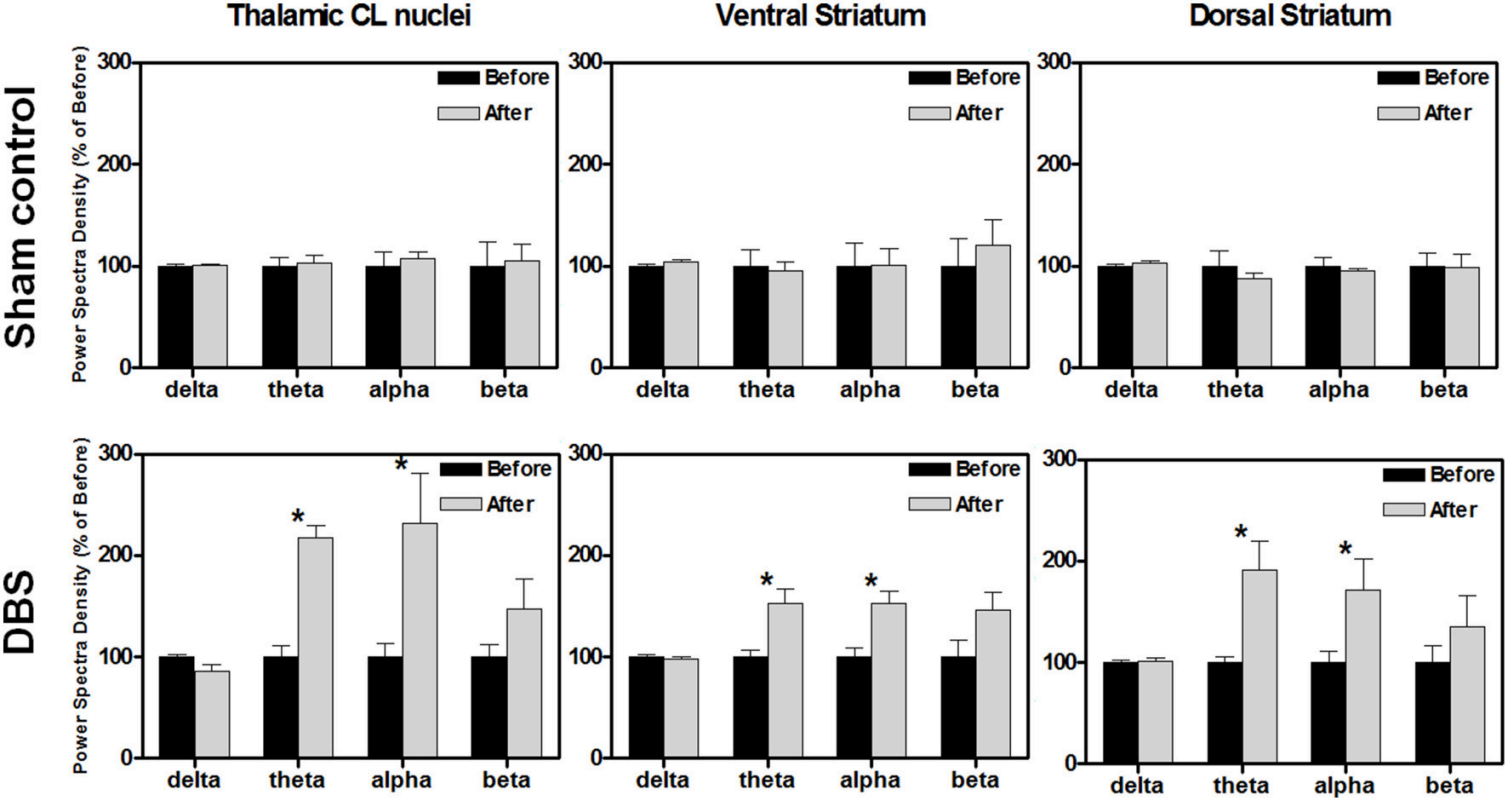
**The comparison % of the power spectral density (PSD) changes of the central lateral thalamic nucleus (CL), ventral striatum (Vstr), and dorsal striatum (Dstr), before and after the water reward-related lever-pressing learning in the sham control group (upper row) and DBS group (lower row), respectively**. The calculated PSD changes of the CL, Vstr, and Dstr were the average of bilateral-channel recordings. The symbol ^*^indicates significant different means with *P* < 0.0125 compared with the respective sham control, and analyzed by Bonferroni correction for multiple comparisons, *N* = 10. Mean ± SEM%.

The neuronal activities of the stimulated brain regions might have directly participated in the enhancement of reward-related lever-pressing learning, so we also recorded the LFP signals from the CL, Vstr, and Dstr, which have been shown to be associated with reward-related learning in rats after behavioral tasks. The oscillations, including delta, theta, alpha and beta were examined. There were no significant PSD differences in the CL, Vstr, and Dstr between before and after the reward-related lever-pressing learning in the sham control group as shown in the upper row of Figure 2 (^*^P > 0.0125, Wilcoxon signed-rank tests with a Bonferroni correction, *N* = 10).

In the DBS-treated group, the statistical analysis revealed that the theta and alpha bands in the CL robustly increased to a level of 236 ± 42% (^*^P < 0.0125, Wilcoxon signed-rank tests with a Bonferroni correction, *N* = 10) and 260 ± 88% (^*^P < 0.0125, Wilcoxon signed-rank tests with a Bonferroni correction, *N* = 10), respectively, compared with those data before reward-related lever-pressing learning (the lower row of Figure 2). Therefore, enhancement of theta and alpha oscillations in the CL, Vstr, and Dstr might be highly associated with reward-related lever-pressing behavior (instrumental skill learning). Detailed PSD traces from the sham control and CT-DBS groups are shown in the Figure S2.

**Figure 3:**
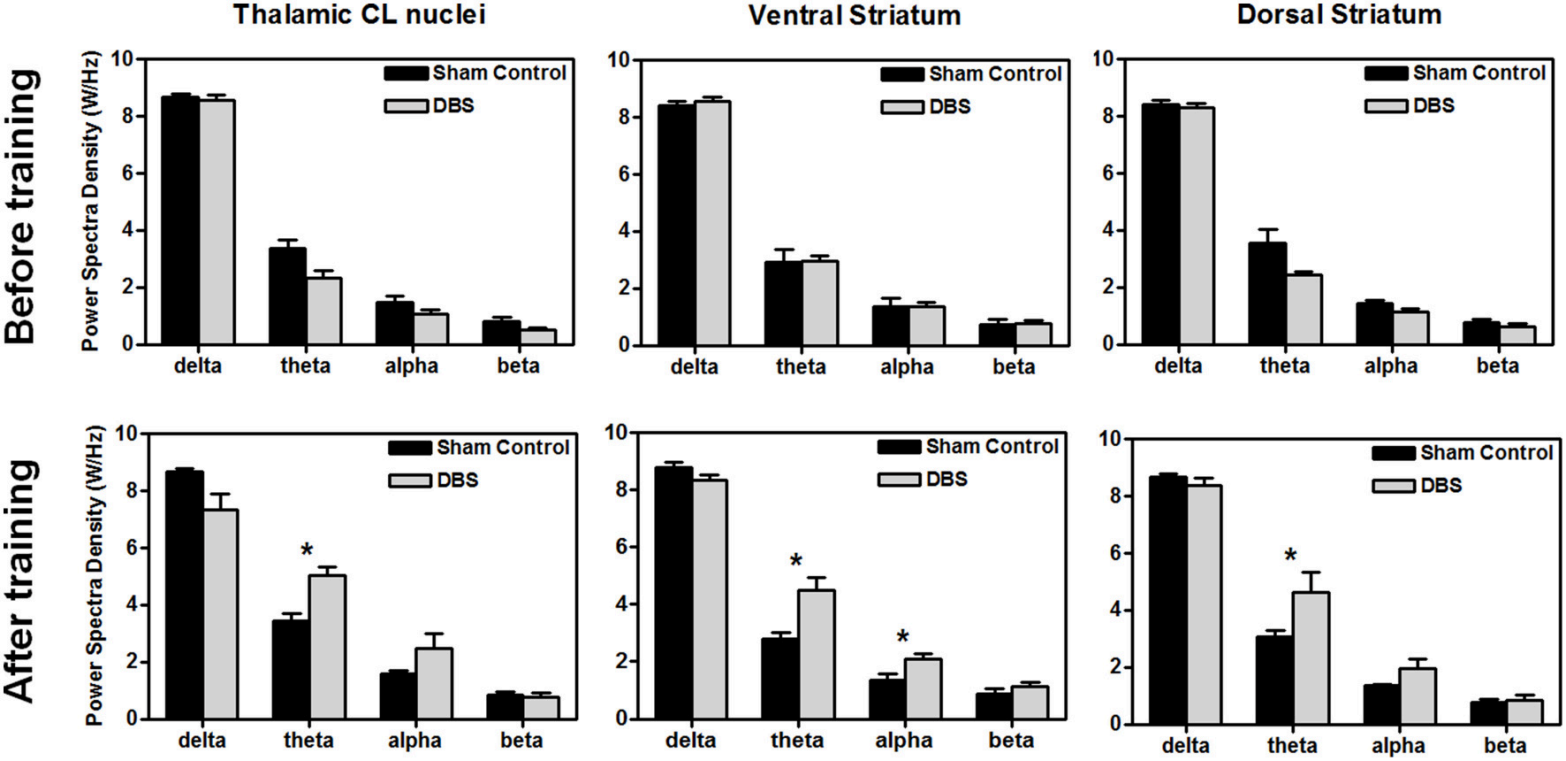
**Comparison of power spectral density (PSD) between groups before (upper row) and after (lower row) the water reward-related lever-pressing learning, respectively**. The calculated PSDs of the central lateral thalamic nucleus (CL), ventral striatum (Vstr), and dorsal striatum (Dstr) were the average of bilateral-channel recordings. The symbol ^*^indicates significant different means with *P* < 0.0125 compared with respect to the sham control (Bonferroni correction for multiple comparisons, *N* = 10). Mean ± SEM.

For comparison of LFP PSD differences between groups after the completion of behavioral testing, statistical analysis of the group data, shown in Figure 3, revealed that DBS treatment altered the amplitude of PSD peaks, and elevated the significantly higher spectral density over the theta band in the CL (^*^P < 0.0125, Wilcoxon two-sample tests with a Bonferroni correction, *N* = 10), and theta and alpha bands in the ventral striatum (^*^P < 0.0125, Wilcoxon two-sample tests with a Bonferroni correction, *N* = 10). There was significant elevation in the theta power after DBS treatment in the dorsal striatum compared with the sham control group (^*^P < 0.0125, Wilcoxon two-sample tests with a Bonferroni correction, *N* = 10). Our results indicate that CT-DBS drastically increases oscillations, especially for the theta band, and might contribute to cognition related learning ability.

### Functional connectivity comparison: CT-DBS vs. sham control

To compare the effect of CT-DBS on functional connectivity among local populations of neurons in the CL, Vstr, and Dstr, we used the coherence between aggregated neuronal activities as an index of functional connectivity. Correlation matrices with delta, theta, alpha, and beta bands, respectively, are shown in Figure S3.

**Figure 4:**
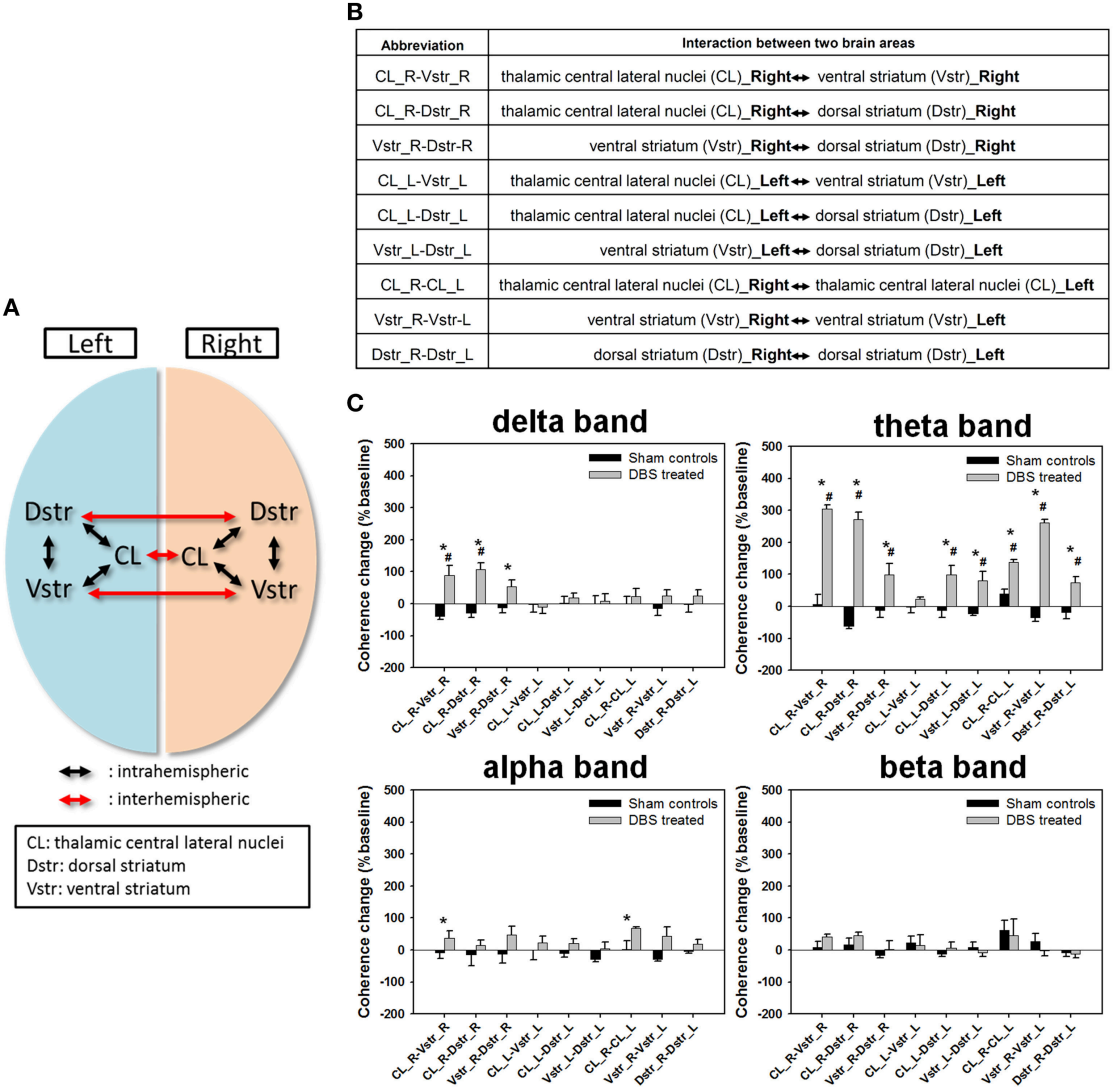
**(A)** The schematics of intrahemispheric and interhemispheric coherences measured between two different brain areas. **(B)** Abbreviation table of intrahemispheric and interhemispheric coherences between two different brain areas. **(C)** Statistical comparison of the coherence changes of the functional connectivity between two recording regions (bilateral central lateral thalamic nucleus [CL], ventral striatum [Vstr,], and dorsal striatum [Dstr]). Data are expressed as means ± SEM%. ^*^P < 0.0125 significant differences in coherence changes between the sham control and DBS-treated groups with the Bonferroni correction for multiple comparisons. ^#^*P* < 0.0125 significant coherence changes compared to baseline between same brain areas with the Bonferroni correction for multiple comparisons.

Further, we examined the changes in inter- and intra-hemispheric coherences across six brain areas (Figures 4A,B), where normalized synchronization changes were quantified between LFP channel-pairs at various frequency bands. The comparison shown in Figure 4C illustrated that the coherence changes of the CT-DBS group in delta band were significantly increased in the right hemispheric△Coh^intra^(CL−Vstr)%, △Coh^intra^(CL−Dstr)%, and △Coh^intra^(Vstr−Dstr)%, as compared with the sham control group. In addition, most of the coherence changes in the theta band were largely increased except for the left hemispheric △Coh^intra^(CL−Vstr). Moreover, the coherence changes in the alpha band were significantly increased in the right hemispheric △Coh^intra^(CL−Vstr)% and inter-hemispheric △Coh^inter^(CL−CL). However, there was no significant coherence change in the beta band. The more detailed description for the comparison of functional connectivity of the paired brain areas was in the Supplementary Note.4.

### C-Fos expression comparison: CT-DBS vs. sham control

**Figure 5:**
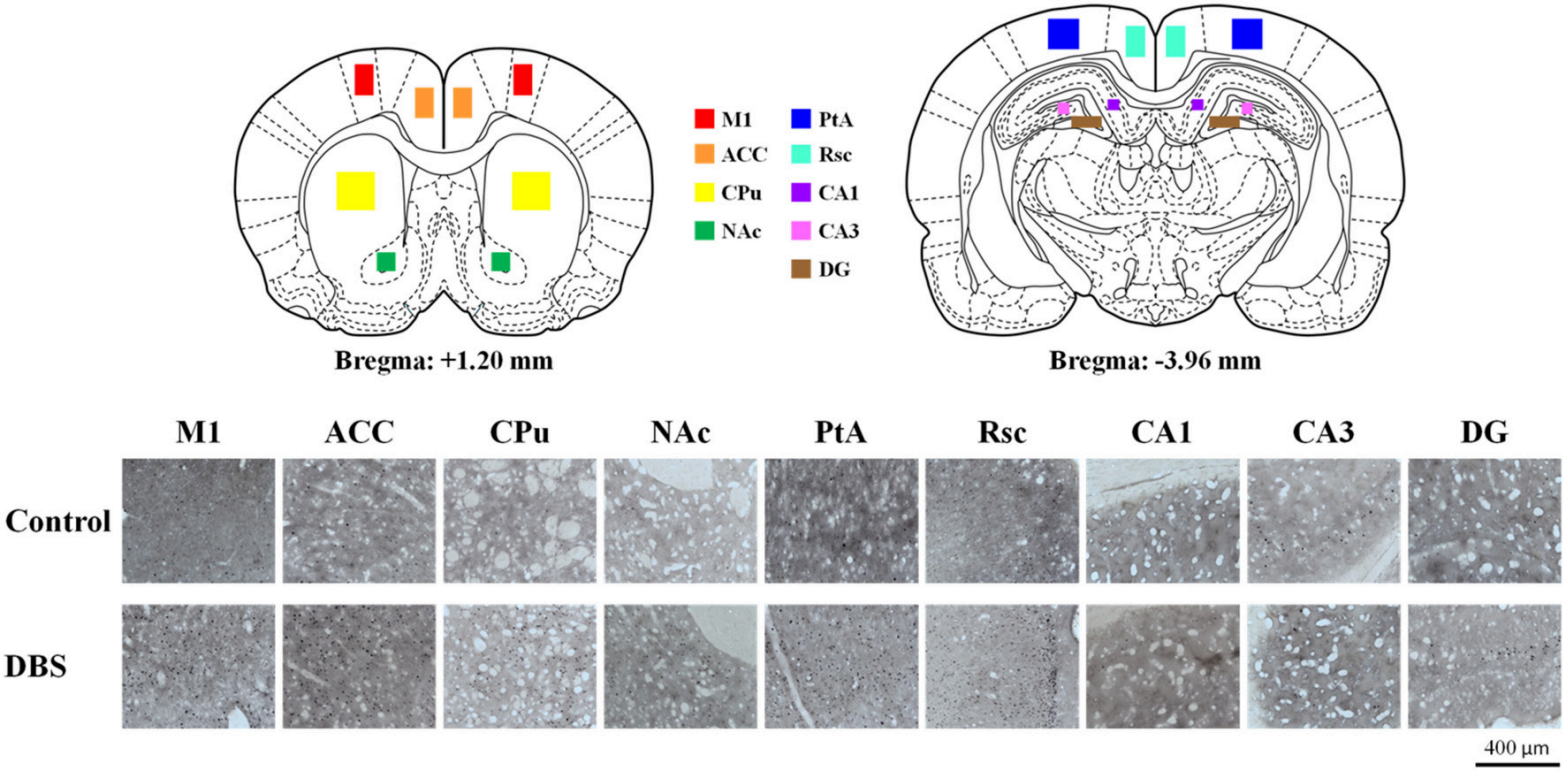
***C-Fos* expression was up-regulated by CT-DBS**. The cartoon depicts a representative area of *c-Fos* staining quantification from the nine brain regions where *c-Fos* was counted (primary motor cortex [M1], anterior cingulate cortex [ACC], caudate-putamen in dorsal striatum [CPu], accumbens nucleus in ventral striatum [NAc], retrosplenial cortex [Rsc], parietal association cortex [PtA], and hippocampus [CA1, CA3, and DG]). Representative photomicrographs show the immunostaining for *c-Fos* expression in each brain region from sham control and CT-DBS samples.

The expression of neuronal *c-Fos* is a well-known marker of neuronal activity. After completing the behavioral training, animals treated with CT-DBS (or sham) were held for an additional 30 min and then single housed for 2 h for the peak expression of the *c-Fos* protein after cell activation in the isolated environment (Waters et al., 1997). In the comparison of neuronal activation distribution between CT-DBS and sham control groups, we found enhancement of *c-Fos*-positive cells in the M1, ACC, CPu, Nac, Rsc, PtA and hippocampal CA1, CA3, and DG as illustrated in Figure 5.

**Figure 6:**
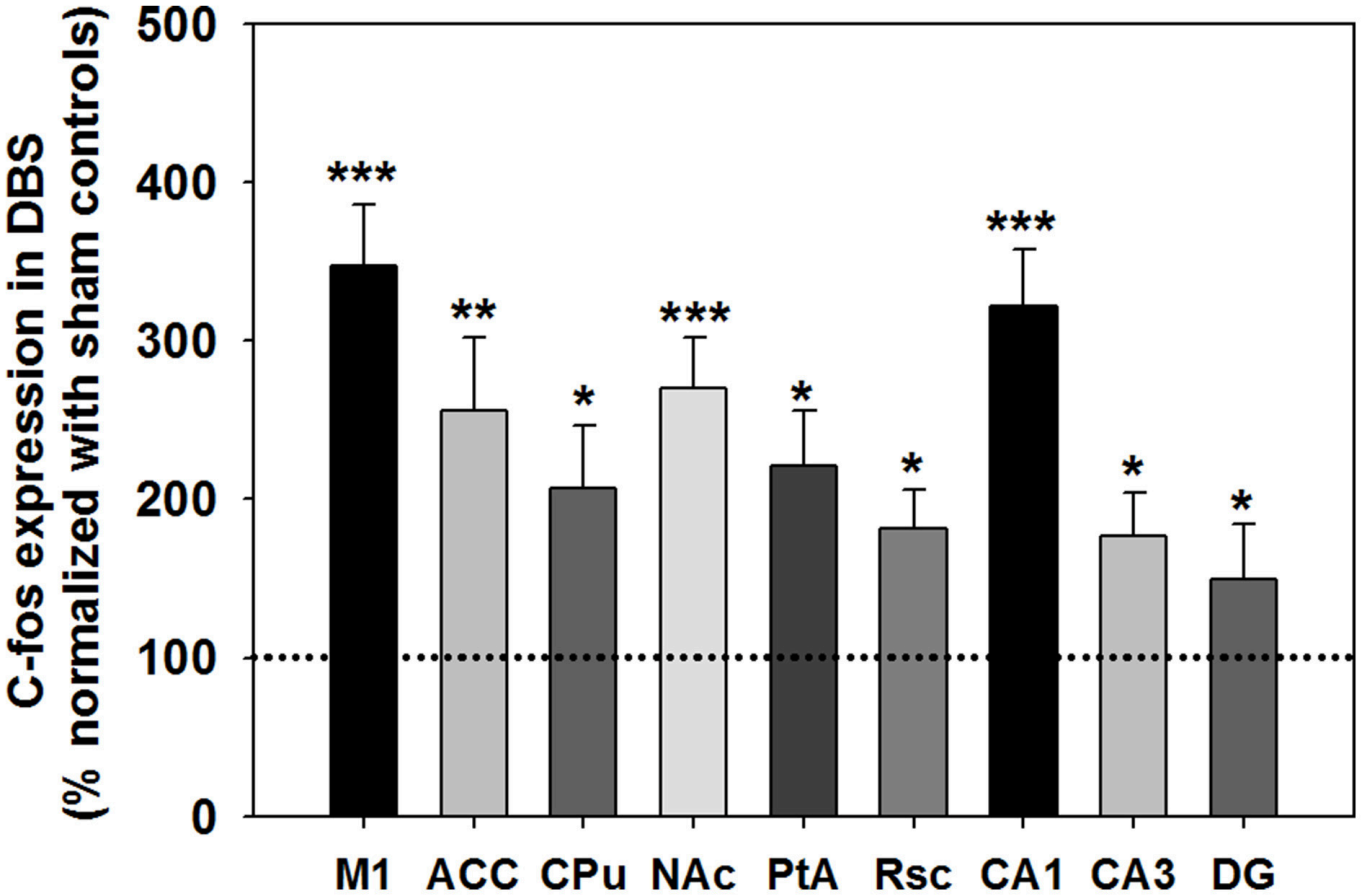
**The *c-Fos* expression after DBS was assessed and normalized to sham control (primary motor cortex [M1], anterior cingulate cortex [ACC], caudate-putamen in the dorsal striatum [CPu], accumbens nucleus in the ventral striatum [NAc], retrosplenial cortex [Rsc], parietal association cortex [PtA], and hippocampus [CA1, CA3 and DG])**. ^*^, ^**^, and ^***^indicate significant *c-Fos* expression with *P* < 0.05, *P* < 0.01, and *P* < 0.001 compared with the sham control, analyzed by Wilcoxon two-sample tests (Mean ± SEM). The dotted line is the baseline of *c-Fos* expression (100%) for the sham control group.

*c-Fos* expression in the analyzed brain areas was normalized and expressed as % compared with the sham control group, as shown in Figure 6. The statistical analysis revealed a significant increase in *c-Fos*-positive neurons in the CT-DBS group relative to the sham control group, observed in the M1 (347 ± 39%; ^***^P < 0.001, Wilcoxon two-sample tests, *N* = 5), ACC (256 ± 46%; ^**^P < 0.01, Wilcoxon two-sample tests, *N* = 5), CPu (207 ± 39%; ^*^P < 0.05, Wilcoxon two-sample tests, *N* = 5), NAc (270 ± 32%; ^***^P < 0.001, Wilcoxon two-sample tests, *N* = 5), Rsc (184 ± 24%; ^*^P < 0.05, Wilcoxon two-sample tests, *N* = 5), PtA (221 ± 35%; ^*^P < 0.05, Wilcoxon two-sample tests, *N* = 5), hippocampal CA1 (322 ± 25%; ^***^P < 0.001, Wilcoxon two-sample tests, *N* = 5), CA3 (167 ± 35%; ^*^P < 0.05, Wilcoxon two-sample tests, *N* = 5), and DG (150 ± 27%; ^*^P < 0.05, Wilcoxon two-sample tests, *N* = 5). Taken together, our results show that CT-DBS significantly increases widespread neuronal activity, however the mechanism of interaction in the activated brain regions, which conveys the cognitive enhancement, needs to be further characterized.

### Dopamine and acetylcholine receptors are up-regulated by CT-DBS

**Figure 7:**
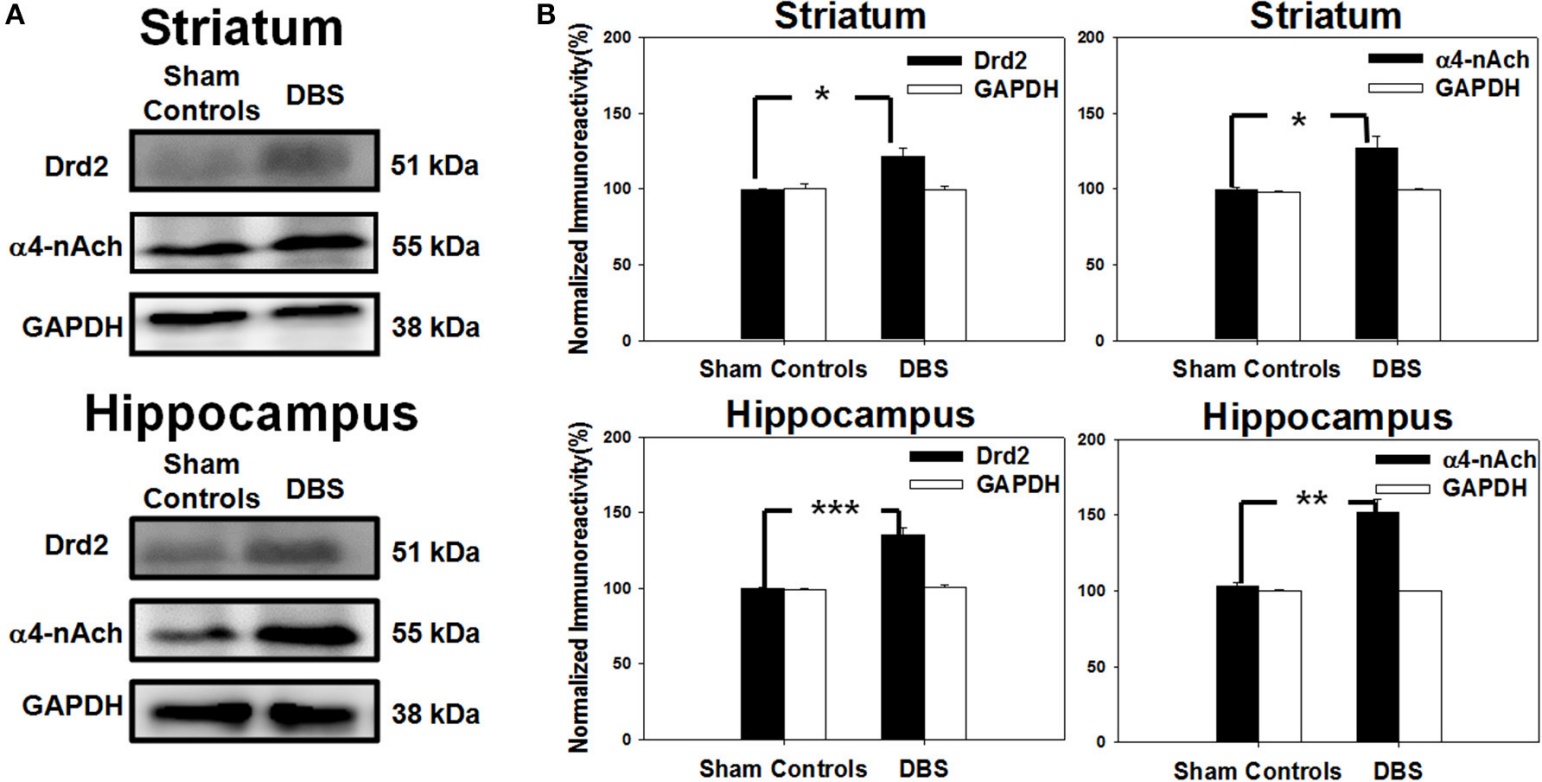
**Western blot protein analysis of cells excised individually from striatal and hippocampal tissues. (A)** The SDS-PAGE blots show the expression of dopamine D2 receptor (Drd2) and α4-nicotinic acetylcholine receptor (α4-nAChR) in the striatum (top) and hippocampus (bottom). **(B)** Results from the quantitative analysis of Drd2 and α4-nAChR expression (mean ± SEM; expressed as ratio to GAPDH) in the striatum (top) and hippocampus (bottom). Striatal and Drd2 and α4-nAChR protein expressions were significantly increased relative to sham control group. Significant increases in Drd2 and α4-nAChR protein expressions in the hippocampus also were found. ^*^, ^**^, and ^***^ indicate significant *protein* expression with *P* < 0.05, *P* < 0.01, and *P* < 0.001, respectively, relative to sham control group.

The striatal neural circuits are composed of dopaminergic and cholinergic synapses that receive signals from the cerebral cortex and propagate them to the basal ganglia to modulate reward-related learning. Thus, we examined the protein levels of Drd2 and α4-nAChR in the striatum by Western blot analysis. In the top row of Figure 7A, the results revealed up-regulation of Drd2 and α4-nAChR in the striatum after DBS treatment compared with sham controls. The statistical analysis indicated that the striatal levels of Drd2 and α4-nAChR protein were significantly increased to 120.3 ± 9.9 and 124.9 ± 10.1%, respectively, compared with the respective sham controls, *N* = 5 (Figure 7B). The levels of hippocampal Drd2 and α4-nAChR protein, as shown in the bottom row of Figure 7A, were also significantly increased to 135.0 ± 5.0 and 152.0 ± 8.8% compared to the respective sham controls, *N* = 5 (Figure 7B). The data demonstrate CT-DBS-induced up-regulation of Drd2 and α4-nAChR receptor expression in the striatum and hippocampus.

## Discussion

Our study demonstrated that electrical stimulation of CT produced a novel enhancement of the water-reward lever-pressing behavior associated with instrumental learning (operant conditioning), the increasing in neural theta oscillations, and the strength of connections of CT with the striatum. Meanwhile, CT-DBS induced widespread *c-Fos* expressions in cortical and subcortical areas since CT widely projected to striatum as well as cortical targets. Significant both Drd2 and α4-nAChR expressions were found in hippocampus and striatum as well.

### CT-DBS enhanced theta LFP oscillation as the biomarker for correlated to instrumental learning

CT-DBS increased both theta and alpha LFP oscillations in CT, Vstr, and Dstr, and largely enhanced water-reward lever-pressing associated with instrumental learning (operant conditioning) as well. Our findings are consistent with a series of studies that alpha and theta LFP oscillations has been potentiated in learning and memory processing of cortical and subcortical regions (Klimesch et al., 1997; Buzsaki, 2002; Knyazev, 2007; Kirov et al., 2009). Consistent with the above proposed neural oscillations for the instrumental learning regulation, Shirvalkar et al. (2006) demonstrated CT-DBS increased generalized arousal and recognition memory performance, such as untrained goal-directed seeking behavior, exploratory motor activity, grooming, and object recognition memory through selective network activation in intact rats. Therefore, CT-DBS enhancement for the water-reward lever-pressing learning was characterized by the prominent theta LFP oscillation, as a reliable biomarker correlated to animal skill learning, recorded throughout the thalamic-striatal neural circuit.

### CT-DBS contribution of connections of CT with the striatum

Our results showed that the significantly increasing intrahemispheric and interhemispheric theta-band coherences in the paired brain areas of CT−Vstr, CT−Dstr in the CT-DBS treated animals. The functional connectivity between the CT and striatum consistent with other reports, including the neuroanatomical mechanisms (Deschenes et al., 1996) and neural signal processing (Yin and Knowlton, 2006) for the modulation of motor control and learning ability. The CT-DBS enhanced local theta-band activities synchronized between distant areas in the thalamic-striatal circuit indicating that excitatory long-range projections functionally coupled CT and striatum connections in this study (Womelsdorf et al., 2010). Meanwhile, our results showed that the CT-DBS modulated the connectivity of Vstr−Dstr with significantly increasing the theta-band coherence, indicating that the Vstr (motor) and Dstr (associative) were both strongly activated simultaneously during the water reward-related skill-learning (Cardinal and Cheung, 2005; Thorn and Graybiel, 2014; Nagel et al., 2015).

In addition, our observation that the alpha- and beta- band coherences in sham control group were slightly decreased without significance in water-reward lever-pressing learning task. During the behavioral task, many factors might influence the changes in strength of the functional connectivity including how thirsty the animal is, how desirable the reward is, or how familiar the animal is with the environment. From animal behavioral video recording in the sham control group, we found animals less actively exploring their environment after reaching the criterion of the behavioral task when compared to the exploratory activity before training. Less rats' exploratory behavior in the late phase could hypothetically reflect any or all of the several internal factors, including most obviously lack of thirst (others include frustration and fatigue). Therefore, the slight decreases in alpha- and beta- band coherences might be due to less reward-motivated behavior toward the tasks in the rats (Sturman and Moghaddam, 2011; Neale et al., 2015). Furthermore, many studies have reported striatal LFPs modulation in the theta band during exploratory behavior (Tort et al., 2008; Lepski et al., 2012). Oscillatory activity was also observed in the delta and beta bands (Hasselmo et al., 2002; Lepski et al., 2012) as well. For functional connectivity, striatal LFPs were also oscillated in strong coherence with the theta rhythm in the prefrontal cortex and thalamus (Ishii et al., 1999; McCracken and Grace, 2009). In the sham control group, animals performed less exploration after the behavioral task, which might be the causes of the slight decrease in the theta-, delta-, and beta- band coherences when compared to the animals before training.

One of the unanticipated findings in our study was that the bilateral CT-DBS increased the coherence change at the delta band in the intra-hemisphere lead to enhancing the lateralized motor skill leaning (the water reward-related lever-pressing learning). To our knowledge, this is the first report of lateralization effect in coherence by bilateral CT-DBS. This effect might be due to that the rats preferred to press the lever by forelimb (handedness; data not shown). However, the function of hemispheric lateralization or asymmetry effect related to CT-DBS increased skill learning needs to be further investigated. Taken together, our results provided evidence that the neuronal activities, especially theta oscillations, in CT and striatum are highly correlated to animal skill learning and modulated by CT-DBS treatment.

### CT-DBS molecularly widespread neuromodulation

DBS is an established therapeutic approach to modulating abnormal neuronal firing of the subthalamic nucleus or internal segments of the globus pallidus for Parkinson's disease patients, whereas several findings have revealed that DBS also functions as a stimulation device to activate the neural network for cognitive functions and alter underlying molecular modification, such as gene expression (Shirvalkar et al., 2006). Accordingly, a well-known activity-dependent neuronal marker, *c-Fos*, is largely up-regulated in several brain regions of CT-DBS rats, especially the Dstr, Vstr, hippocampus, Rsc and PtA. The *c-Fos* up-regulation in the CPu and Dstr, and the NAc in the Vstr demonstrates that CT-DBS activates and increases connectivity between Vstr and Dstr to enhance motor ability and reward-related skill learning. We observed *c-Fos* expression was also elevated among the hippocampus (CA3 and CA1), ACC, Rsc, Pta, and M1. Hippocampus has been demonstrated that it is involved control and process of learning and memory signals (Kesner, 2013). One study revealed that the ACC might regulate cognitive and emotional processing, even motor and sensory functions (Bush et al., 2000). In addition, Rsc and Pta have emerged as key regions of the brain network that supports spatial navigation and short term memory (Maguire, 2001; Vann et al., 2009). Recently finding indicated that the ACC projects axons to the Rsc and PtA, and should be considered an important component related to reward anticipation, decision-making, impulse control, and emotion (Ragozzino and Rozman, 2007). Taken together, the results suggest that CT-DBS causes widespread cerebral network activation that is associated with cognition and motor function by up-regulation of immediate early gene expression.

### CT-DBS enhanced functional synaptic connections associated with Drd2 and α4-nAChR

We further examined the synaptic neurotransmitter mechanisms underlie CT-DBS induced the enhancement of functional connectivity and water-reward level pressing learning. Our data showed the expression of Drd2 and α4-nAChR was increased in striatum and hippocampus following CT-DBS. Previous findings suggested striatum is the main component mediating various inputs of the basal ganglia neural circuit, and the interaction of dopaminergic and cholinergic signaling is crucial for cognitive functions, motor activity, and reward-related information (Calabresi et al., 2006; Cragg, 2006; Calabresi and Di Filippo, 2008). The Drd2 participates in modulating local motor activity, schizophrenia, and working memory in the prefrontal cortex (Luciana et al., 1992; Baik et al., 1995; Wang et al., 2004). Dorsolateral striatum is involved in the initial discrimination of exercise-associated tasks mediated by up-regulation of striatal Drd2 (Eddy et al., 2014). Additionally, activation of α4-nAChR in striatum and hippocampus increased following CT-DBS. Meanwhile, the nicotinic cholinergic system plays a pivotal role in working memory and attention in the hippocampal and prefrontal regions (Levin and Simon, 1998; Ross et al., 2000; Labarca et al., 2001). Thus, we mentioned that dopaminergic and cholinergic systems are the major neurotransmission system in water reward-related skill-learning. Applied of CT-DBS evoked an up-regulation of Drd2 in the stratum, indicating that the enhancement of reward-associated learning might be due to increase activity of the Drd2. This suggests that the inter-regional connectivity enhancement might contribute to synaptic plasticity, at least in the striatum, by altering expression of dopaminergic and cholinergic receptors that modulate striatal synaptic plasticity to regulate downstream signaling cascades for higher brain reward-related skill learning process.

This study suggested that DBS at CT modulates a cortical network for reward-related skill-learning behavior. CT-DBS is seen to produce increase of oscillation patterns and functional connectivity of thalamus-striatum network, especially in the theta band. We also delineated a possible underlying molecular mechanism that involves activation of neuronal projections from the CL to striatal dopaminergic neurons and up-regulation of the *c-Fos*, Drd2, and α4-nAChR of the striatum to modulate higher cognitive learning function. Our future studies will further explore the function of these circuits in small animal model with neurodegenerative diseases.

## Author contributions

HCL, HYL, and YYC designed the project, organized the entire research. SL, HP, and YL conceived the experiments. HP, YL, ES, LL, PL, YWC, and YYC conducted the experiments. HCP, YL, YYC, KL, and FJ analyzed the results. HCL, HYL, and KC performed the immunohistochemistry and Western blot studies. HCL, ES, HYL, and YYC wrote the manuscript. All authors discussed the results and reviewed on the manuscript.

### Conflict of interest statement

The authors declare that the research was conducted in the absence of any commercial or financial relationships that could be construed as a potential conflict of interest.
