# Supplementary material for: Central Thalamic Deep-Brain Stimulation Alters Striatal-Thalamic Connectivity in Cognitive Neural Behavior
Source: Front Neural Circuits. 2016 Jan 13;9:87. doi: 10.3389/fncir.2015.00087 (PMC4710746; doi:10.3389/fncir.2015.00087)
Supplement: Supplementary file 2 [file DataSheet1.DOCX]

Supplementary Material

Altered Striatal–Thalamic Connectivity in Relation to Cognitive Neurobehavior with Central Thalamic Deep-Brain Stimulation

Hui-Ching Lin^1,2, +^ , Han-Chi Pan^3, +^, Sheng-Huang Lin,^4,5^ Yu-Chun Lo^6^, Elise Ting-Hsin Shen^5^, Lun-De Liao^7,8^, Pei-Han Liao^9^, Yi-Wei Chien^89^, Kuei-Da Liao^10^, Fu-Shan Jaw^5^, Hsin-Yi Lai^11,*^ and You-Yin Chen^9,*^

^*^Correspondence should be addressed to either of the following:

Dr. Hsin-Yi Lai, Interdisciplinary Institute of Neuroscience and Technology, Qiushi Academy for Advanced Studies, Zhejiang University, No.268, Kaixuan Rd., Hangzhou, Zhejiang, China, 310029

Email: laihy@zju.edu.cn

Dr. You-Yin Chen, Department of Biomedical Engineering, National Yang Ming University, No.155, Sec.2, Linong St., Taipei, Taiwan 112, ROC

E-mail: irradiance@so-net.net.tw

*Note 1. Location of electrodes implanted in vivo*


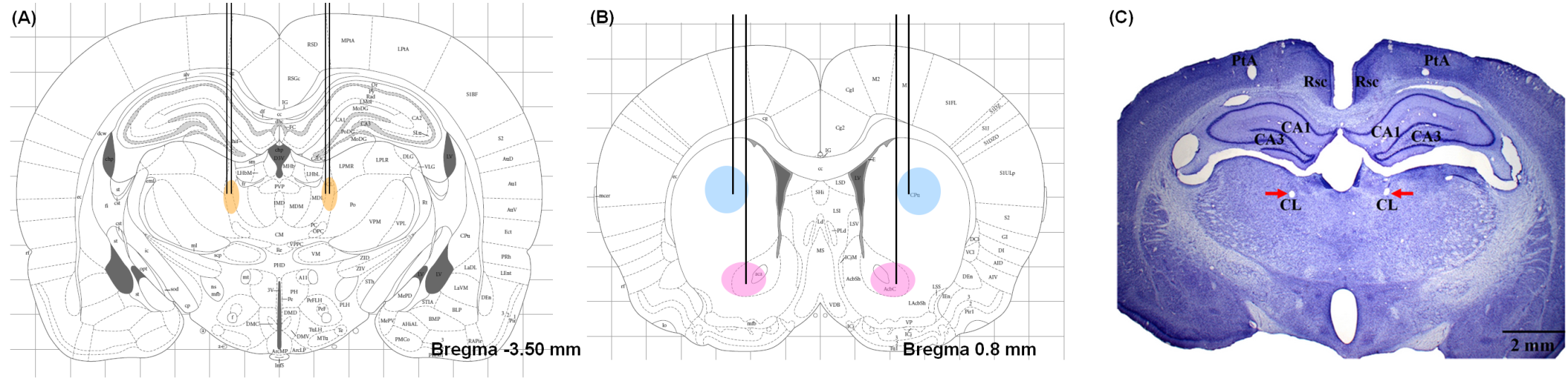


**Figure S1**. (A) Schematic representation of two-paired microwire electrode placements in the bilateral central lateral thalamic nucleus (CL). One paired microwire electrodes on each side was used to perform central thalamic deep brain stimulation (CT-DBS) and LFP recording. (B) Schematic depiction of two-paired microwire electrode placements in the bilateral striatum. Two microwire electrodes on each side were used to perform LFP recordings in Vstr and Dstr, respectively. (C) Photomicrograph of a Nissl-stained coronal section at 3.5 mm posterior to bregma. CL: central lateral thalamic nucleus; CA1 and CA3: hippocampus; Rsc: retrosplenial cortex; PtA: parietal association cortex. Reference lesion sites (red arrowheads) were observed bilaterally in the CL, as indicated by the microwire electrode location.

*Note 2. Frequency spectrum analysis*

All multichannel local field potentials (LFPs) were first band-pass filtered at 0.5-55 Hz with a 2^nd^ order IIR Butterworth digital filter, and then down sampled at 200 Hz and sliced into 10 s windows. The power spectral density (PSD) of the LFP was computed using fast Fourier transform (FFT), with the Welch spectral estimator and a Hamming window of 2 s with 50% overlap and 1 Hz resolution ([Buzsáki, Anastassiou, & Koch, 2012](#_ENREF_1); [Jia, Smith, & Kohn, 2011](#_ENREF_2)). Each recording session was detrended to remove any slow DC components and padded with zeros to increase frequency resolution. PSD of LFP oscillations in each region of the DBS (or sham control) group was calculated as compared to those of the baseline. There were no significant peaks found in the PSD of LFP occurring in the central lateral thalamic nucleus (CL), ventral striatum (Vstr), and dorsal striatum (Dstr) in the sham control group (upper panel in the **Figure S2**). In the DBS-treated group, spectral analysis of the LFP revealed two PSD peaks at theta and alpha bands in the CL and Dstr, and three significant peaks at theta, alpha, and beta bands in the ventral striatum (lower panel in the **Figure S2**).


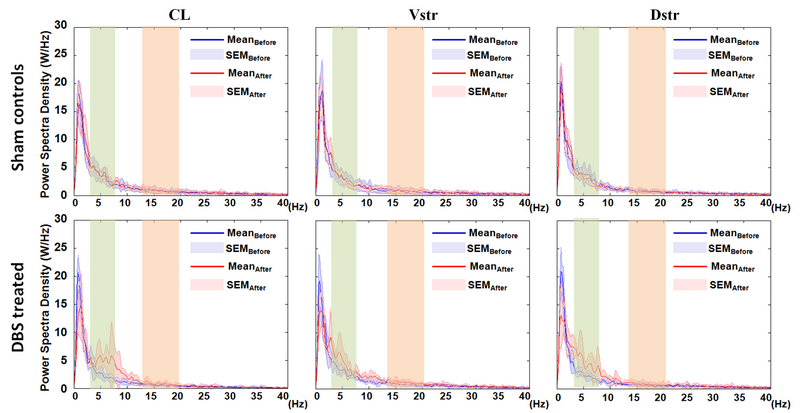


**Figure S2**. Central thalamic deep brain stimulation (CT-DBS) increased LFP oscillation theta and alpha bands of the central lateral thalamic nucleus (CL), ventral striatum (Vstr), and dorsal striatum (Dstr). The PSDs of the CL, Vstr, and Dstr are shown. The blue line is the mean spectrum of the baseline, before completing the behavioral task, and the red line is the mean spectrum after completing the behavioral task.

*Note 3. Coherence spectral estimates*

Coherence is a measurement of linear association (correlation) between two signals across frequencies. The magnitude squared coherence$C_{xy}(f)$ was calculated by means of the multitaper method ([Jia et al., 2011](#_ENREF_2); [Pesaran, Pezaris, Sahani, Mitra, & Andersen, 2002](#_ENREF_3)):

$C_{xy}(f)= \frac{\left| P_{xy}(f) \right|^{2}}{P_{xx}\left( f \right)P_{yy}(f)}$ (S1)

where $P_{xx}(f)$and $P_{yy}(f)$ are the averages of the spectral powers of the LFP time series and $P_{xy}(f)$ is the average cross-spectral power. The magnitude squared coherence values for each channel pair were computed using Welch's method, a modified periodogram method. The magnitude coherence estimate is a function of frequency with values ranging from zero to one, where zero coherence means that the LFPs are unrelated, and a coherence of one means that the LFPs have a constant phase relationship. Data were analyzed offline using custom-built MATLAB software (MATLAB R12, Mathworks Inc., USA). Magnitude squared coherence (**function** "**mscohere**" in MATLAB) was used in this study. Magnitude squared coherence measurement parameters include coherence frequency segment size (5000 points), Hanning window overlap (50%) and tapering, and sampling rate (1 kHz). We found a strong delta and theta coherence increases between the CL and striatum in the DBS-treated group as shown in **Figure S3**. Thus, CL/striatum coherence could lead to the synchronization of reward-predicting activity in the prefrontal networks, tagging it for subsequent memory consolidation.


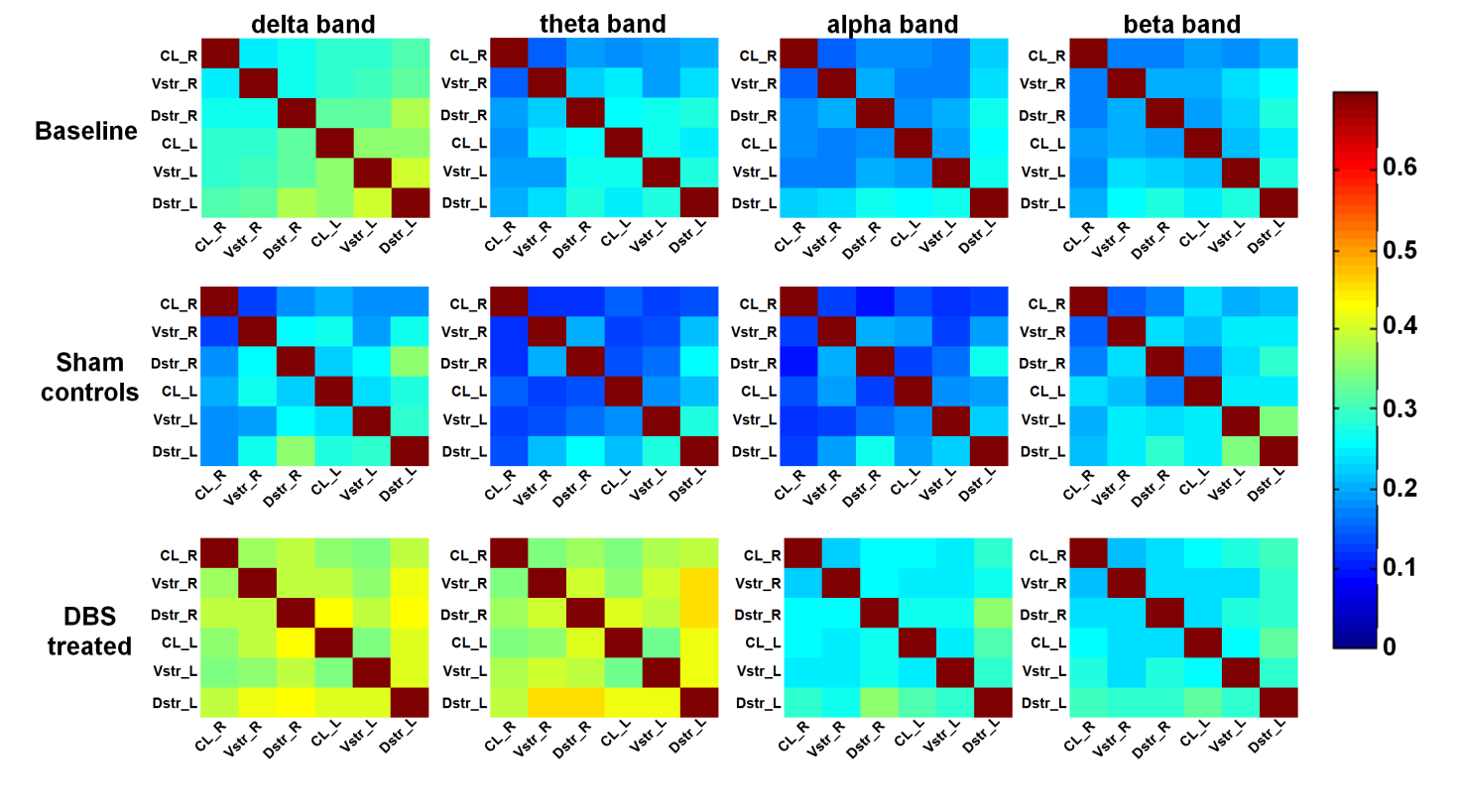


**Figure S3**. Comparison of the cross-spectral density matrices in the frequency domain of the functional connectivity among baseline (before behavioral training), sham control, and DBS-treated groups (after behavioral training). The correlation matrices obtained showed a strong bilateral correlation of the delta and theta synchronizations in the central lateral thalamus nucleus (CL), ventral striatum (Vstr), and dorsal striatum (Dtsr), as well as between the CL and striatum. Other frequency synchronizations, such as correlation matrices in the alpha and beta bands showed weak-to-no correlation between the CL and striatum.

*Note 4.* *Comparison of functional connectivity in the paired brain areas*

The comparison shown in **Figure 4**C illustrated that in DBS-treated animals, the right hemispheric $\Delta\mathrm{Coh}^{\mathrm{intra}}\left( \mathrm{CL}─\mathrm{Vstr} \right)\%$ was significantly increased in the delta band (88.4 ± 18.0%; ^*^*P* < 0.0125, Wilcoxon two-sample tests with a Bonferroni correction), theta band (305.9 ± 8.5%; ^*^*P* < 0.0125, Wilcoxon two-sample tests with a Bonferroni correction) and alpha band (40.0 ± 17.7%; ^*^*P* < 0.0125, Wilcoxon two-sample tests with a Bonferroni correction) as compared to those in the sham controls (delta band: -39.4 ± 7.5%; theta band: 5.8 ± 20.0%; alpha band: -8.4 ± 13.7%).

In comparisons of the right hemispheric $\Delta\mathrm{Coh}^{\mathrm{intra}}\left( \mathrm{CL}─\mathrm{Dstr} \right)\%$ between groups, the LFP synchronizations in the DBS-treated group significantly increased in the delta (106.3 ± 15.2% *verse* -29.6 ± 10.6%; ^*^*P* < 0.0125, Wilcoxon two-sample tests with a Bonferroni correction), and theta (272.4 ± 17.9% *versus* -60.9 ± 7.4%; ^*^*P* < 0.01, Wilcoxon two-sample tests with a Bonferroni correction) bands. Comparing the $\Delta\mathrm{Coh}^{\mathrm{intra}}\left( \mathrm{Dstr}─\mathrm{Vstr} \right)\%$ in the right hemisphere of the rat brain between groups, DBS treatment caused significant increases in delta (54.4 ± 14.6% *versus* -12.0 ± 11.3%; ^*^*P* < 0.05, Wilcoxon two-sample tests with a Bonferroni correction) and theta (98.7 ± 24.8% *versus* -13.0 ±17.6%; ^*^*P* < 0.05, Wilcoxon two-sample tests with a Bonferroni correction) bands.

The left hemispheric$\Delta\mathrm{Coh}^{\mathrm{intra}}\left( \mathrm{CL}─\mathrm{Vstr} \right)\%$, $\Delta\mathrm{Coh}^{\mathrm{intra}}\left( \mathrm{CL}─\mathrm{Dstr} \right)\%$ and $\Delta\mathrm{Coh}^{\mathrm{intra}}\left( \mathrm{Vstr}─\mathrm{Dstr} \right)\%$ after DBS treatment appeared significantly higher for the theta band compared to those of the sham controls (^*^*P* < 0.0125, Wilcoxon two-sample tests with a Bonferroni correction).

For comparison of $\Delta\mathrm{Coh}^{\mathrm{inter}}\left( \mathrm{CL}─\mathrm{CL} \right)\%$ between groups, significant increases in both theta (139.0 ± 5.0% *versus* 39.7 ± 11.5%) and alpha (67.0 ± 3.6% *versus* 2.8 ± 14.8%) rhythms were found in the bilateral CL─CL after DBS treatment (^*^*P* < 0.0125, Wilcoxon two-sample tests with a Bonferroni correction). Meanwhile, DBS treatment resulted in statistically significant increases in the theta rhythm for $\Delta\mathrm{Coh}^{\mathrm{inter}}\left( \mathrm{Vstr}─\mathrm{Vstr} \right)\%$ (44.0 ± 22.2% *versus* -27.7± 5.0%; ^*^*P* < 0.0125, Wilcoxon two-sample tests with a Bonferroni correction) and $\Delta\mathrm{Coh}^{\mathrm{inter}}\left( \mathrm{Dstr}─\mathrm{Dstr} \right)\%$ (18.9 ± 9.4% *versus* -4.6 ± 4.1%; ^*^*P* < 0.0125, Wilcoxon two-sample tests with a Bonferroni correction).

Compared to baseline, the increased delta and theta bands in the right hemispheric $\Delta\mathrm{Coh}^{\mathrm{intra}}\left( \mathrm{CL}─\mathrm{Vstr} \right)\%$ and $\Delta\mathrm{Coh}^{\mathrm{intra}}\left( \mathrm{CL}─\mathrm{Dstr} \right)\%$ were both statistically significant in the DBS-treated group (^#^*P* < 0.0125, Wilcoxon two-sample tests with a Bonferroni correction). Furthermore, we only found the DBS-treated theta band of the right hemispheric $\Delta\mathrm{Coh}^{\mathrm{intra}}\left( \mathrm{Vstr}─\mathrm{Dstr} \right)\%$ was significantly higher than baseline (^#^*P* <0.05, Wilcoxon two-sample tests with a Bonferroni correction). The DBS-treated theta band of the left hemispheric$\Delta\mathrm{Coh}^{\mathrm{intra}}\left( \mathrm{CL}─\mathrm{Dstr} \right)\%$ and $\Delta\mathrm{Coh}^{\mathrm{intra}}\left( \mathrm{Vstr}─\mathrm{Dstr} \right)\%$ were significantly higher than baseline (^#^*P* <0.0125, Wilcoxon two-sample tests with a Bonferroni correction).

For comparisons of$\Delta\mathrm{Coh}^{\mathrm{inter}}\left( \mathrm{CL}─\mathrm{CL} \right)\%$ ,$\Delta\mathrm{Coh}^{\mathrm{inter}}\left( \mathrm{Vstr}─\mathrm{Vstr} \right)\%$ and $\Delta\mathrm{Coh}^{\mathrm{inter}}\left( \mathrm{Dstr}─\mathrm{Dstr} \right)\%$ before (baseline) and after reward-related lever-pressing learning, the DBS-treated theta band (^#^*P* < 0.0125, Wilcoxon two-sample tests with a Bonferroni correction) were significantly different from baseline measurements.

Reference

Buzsáki, György, Anastassiou, Costas A., & Koch, Christof. (2012). The origin of extracellular fields and currents — EEG, ECoG, LFP and spikes. *Nat Rev Neurosci, 13*(6), 407-420. doi: <http://www.nature.com/nrn/journal/v13/n6/suppinfo/nrn3241_S1.html>

Jia, X., Smith, M. A., & Kohn, A. (2011). Stimulus selectivity and spatial coherence of gamma components of the local field potential. *J Neurosci, 31*(25), 9390-9403. doi: 10.1523/jneurosci.0645-11.2011

Pesaran, Bijan, Pezaris, John S., Sahani, Maneesh, Mitra, Partha P., & Andersen, Richard A. (2002). Temporal structure in neuronal activity during working memory in macaque parietal cortex. *Nat Neurosci, 5*(8), 805-811. doi: <http://www.nature.com/neuro/journal/v5/n8/suppinfo/nn890_S1.html>
