# Supplementary figures and images for: Central Thalamic Deep-Brain Stimulation Alters Striatal-Thalamic Connectivity in Cognitive Neural Behavior
Source: Front Neural Circuits. 2016 Jan 13;9:87. doi: 10.3389/fncir.2015.00087 (PMC4710746; doi:10.3389/fncir.2015.00087)

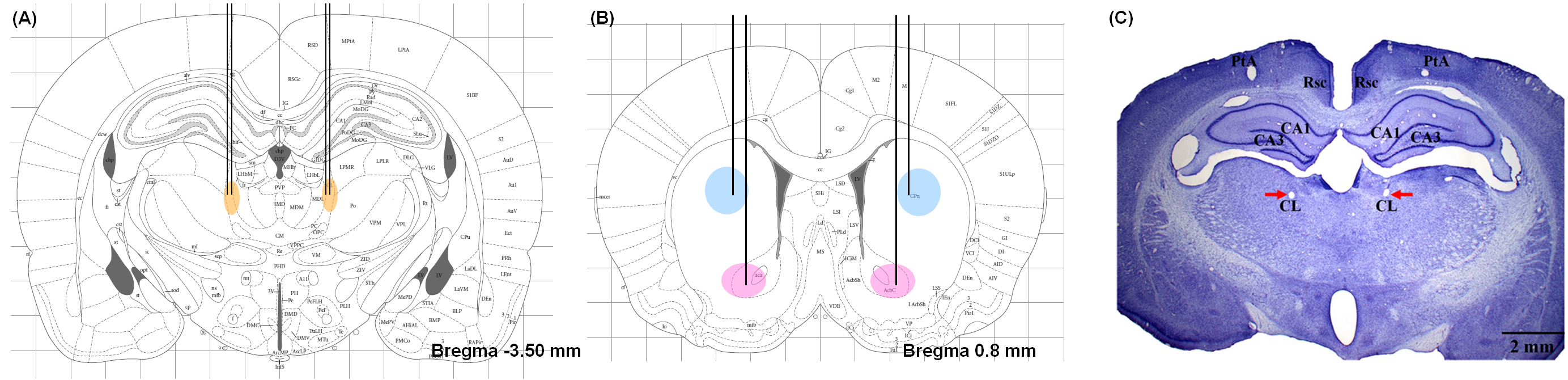

Supplement: Supplementary file 3 [file Image1.TIF]

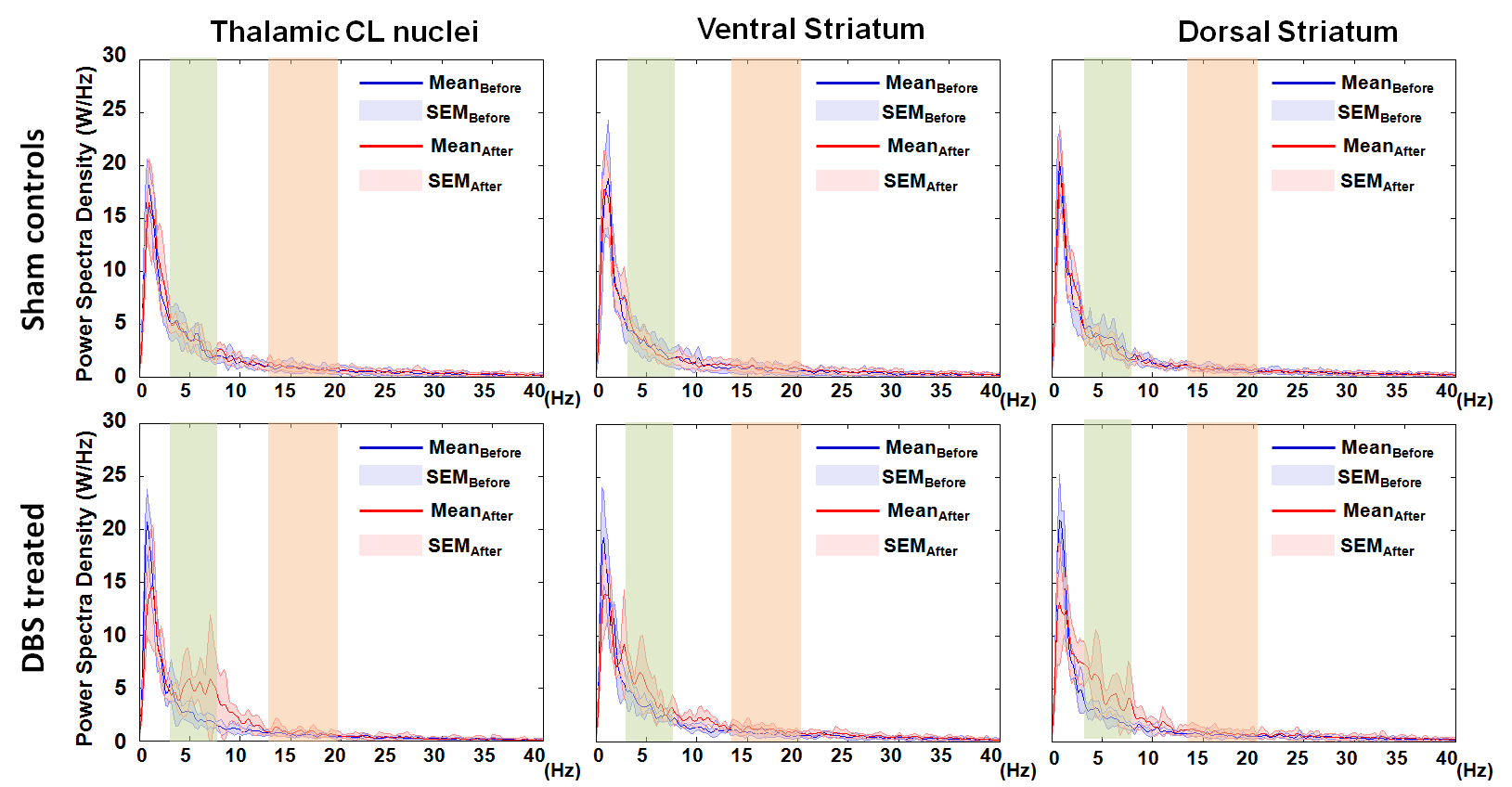

Supplement: Supplementary file 4 [file Image2.TIF]

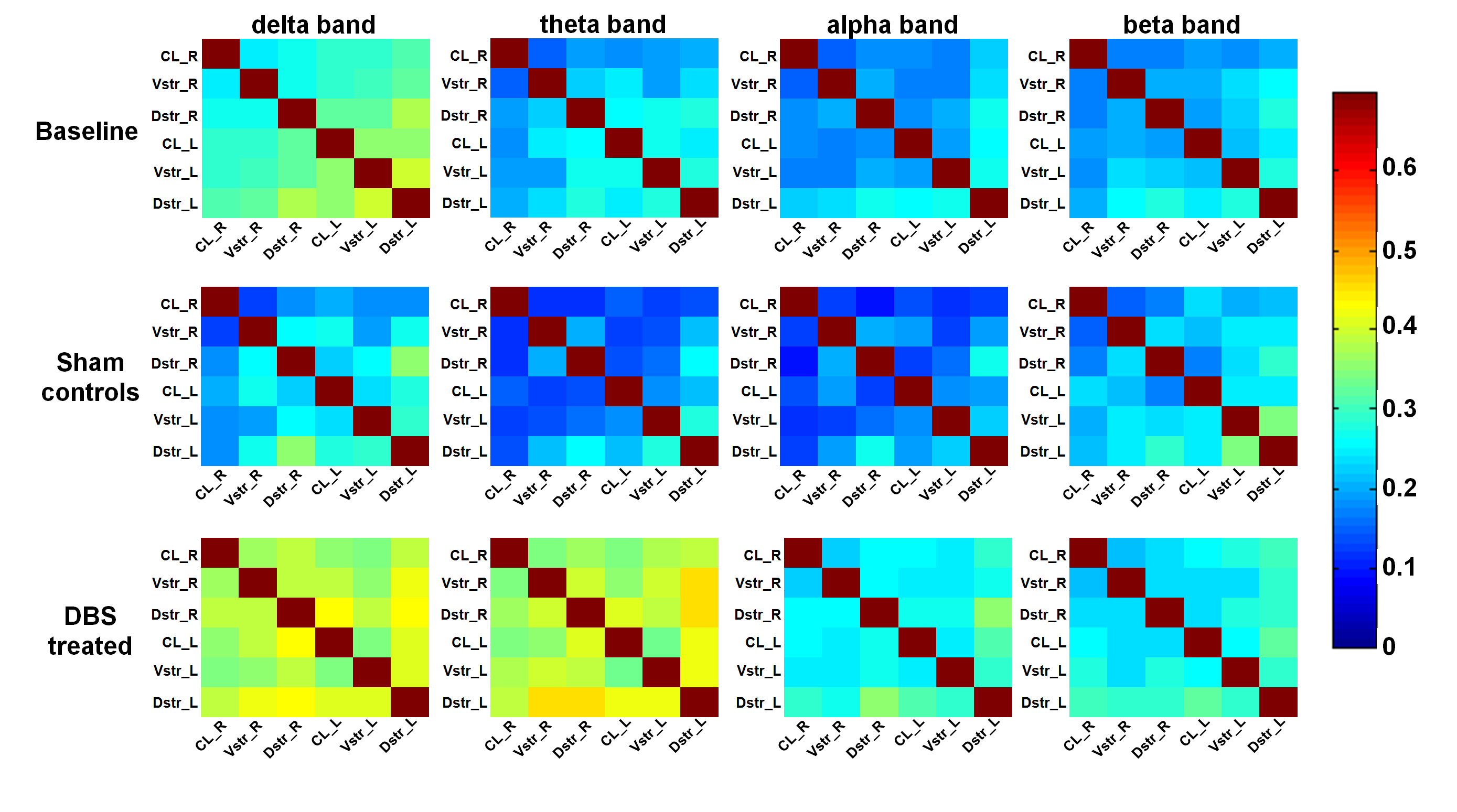

Supplement: Supplementary file 5 [file Image3.TIF]
